# Auditory Proprioceptive Integration: Effects of Real-Time Kinematic Auditory Feedback on Knee Proprioception

**DOI:** 10.3389/fnins.2018.00142

**Published:** 2018-03-08

**Authors:** Shashank Ghai, Gerd Schmitz, Tong-Hun Hwang, Alfred O. Effenberg

**Affiliations:** Institute of Sports Science, Leibniz University Hannover, Hannover, Germany

**Keywords:** perception, rehabilitation, sonification, coordination, joint position sense

## Abstract

The purpose of the study was to assess the influence of real-time auditory feedback on knee proprioception. Thirty healthy participants were randomly allocated to control (*n* = 15), and experimental group I (15). The participants performed an active knee-repositioning task using their dominant leg, with/without additional real-time auditory feedback where the frequency was mapped in a convergent manner to two different target angles (40 and 75°). Statistical analysis revealed significant enhancement in knee re-positioning accuracy for the constant and absolute error with real-time auditory feedback, within and across the groups. Besides this convergent condition, we established a second divergent condition. Here, a step-wise transposition of frequency was performed to explore whether a systematic tuning between auditory-proprioceptive repositioning exists. No significant effects were identified in this divergent auditory feedback condition. An additional experimental group II (*n* = 20) was further included. Here, we investigated the influence of a larger magnitude and directional change of step-wise transposition of the frequency. In a first step, results confirm the findings of experiment I. Moreover, significant effects on knee auditory-proprioception repositioning were evident when divergent auditory feedback was applied. During the step-wise transposition participants showed systematic modulation of knee movements in the opposite direction of transposition. We confirm that knee re-positioning accuracy can be enhanced with concurrent application of real-time auditory feedback and that knee re-positioning can modulated in a goal-directed manner with step-wise transposition of frequency. Clinical implications are discussed with respect to joint position sense in rehabilitation settings.

## Introduction

Real-time kinematic auditory feedback can be effective in enhancing motor perception, control, and learning (Effenberg, 2005, 2014; Sigrist et al., 2015; Effenberg et al., 2016; Dyer J. et al., 2017). The perception of additional real-time acoustic feedback driven by dynamic or kinematic movement parameters obviously supports sensory/perceptual-motor representations (Effenberg, 2005; Schmitz et al., 2013) by enhancing cross-modal stimulation (Scholz et al., 2015; Ghez et al., 2017), multisensory integration (Schmitz et al., 2013; Effenberg et al., 2016), internal motor simulation (Schmitz and Effenberg, 2017), and neural plasticity (Altenmüller et al., 2009; Ghai et al., 2017c). Literature indicates strong associations between auditory and motor areas for enhancing the performance in music (Lahav et al., 2013), breathing (Murgia et al., 2016), writing (Effenberg et al., 2015; Danna and Velay, 2017), sports (Sigrist et al., 2013, 2015; Effenberg et al., 2016), and rehabilitation (Altenmüller et al., 2009; Murgia et al., 2015; Pau et al., 2016; Scholz et al., 2016; Ghai et al., 2017c; Mezzarobba et al., 2017). Strong auditory motor couplings have also been confirmed in neuroimaging studies, where enhanced activation in cortical and sub-cortical structures associated with biological motion perception were reported (Scheef et al., 2009; Schmitz et al., 2013). Several underlying theories have been suggested to ascertain the beneficial effects of concurrent auditory feedback on motor performance. For instance, the concurrent auditory feedback is thought to amplify the brain's ability to integrate multiple congruent perceptual streams, leading to formation of stable internal feed-forward models (Wolpert and Miall, 1996; Calvert et al., 2000; Shams and Seitz, 2008; Van Vugt, 2013). Moreover the real-time availability of feedback can serve as an external guidance for motor execution (Dyer J. F. et al., 2017) as well as an error feedback (Altenmüller et al., 2009; van Beers, 2009; Sigrist et al., 2015; van Vugt and Tillmann, 2015), and can enhance motor imagery (Sigrist et al., 2013), cognitive-emotional functioning (Eschrich et al., 2008; Sihvonen et al., 2017; see also Sigrist et al., 2013).

A strong influence of real-time auditory feedback on motor performance (Eriksson and Bresin, 2010; Schmitz et al., 2014; Scholz et al., 2015; Sigrist et al., 2015; Danna and Velay, 2017; Dyer J. F. et al., 2017), indicates a proportional influence of auditory domain over proprioception (Pantev et al., 2001; Scholz et al., 2015; Effenberg et al., 2016; Danna and Velay, 2017; Sihvonen et al., 2017), and it becomes effective as an integral component of motor control and coordination process (Proske, 2005; Ghai et al., 2017a). Scholz et al. (2015) mentioned that spatio-temporal associations generated by real-time kinematic auditory feedback during motor execution might allow substitution of proprioceptive deficits, possibly by closing the sensorimotor loop (Altenmüller et al., 2009; Särkämö et al., 2016; Scholz et al., 2016). Dyer J. et al. (2017) and van Vugt and Tillmann (2015) further added that the concurrent auditory feedback might supplement the low temporal-perceptual resolution of the proprioceptive domain (Tinazzi et al., 2002). Danna and Velay (2017) in their recent study proposed auditory-proprioceptive substitution for the enhancements the authors reported in handwriting performance for deafferented subjects receiving concurrent auditory feedback. These findings draw inferences from literature pertaining to cross-modal stimuli processing (Stein and Meredith, 1993; Calvert, 2001; Bavelier and Neville, 2002). For instance, sensory convergence from different sensory modalities have been reported to provoke cross-modal interactions (Macaluso et al., 2000; Macaluso and Driver, 2001). Furthermore, these claims are supported by neuroanatomical studies, reporting the presence of long range cortico-cortical connections in between sensory cortices (Falchier et al., 2001; Foxe, 2009; Keniston et al., 2010; Butler et al., 2012), and multisensory integration sites (Chabrol et al., 2015; for a detailed review see Calvert, 2001). This might suggest the possibility of a level of interdependency that the sensory modalities might share with each other to generate an integrated multimodal percept (Macaluso et al., 2000; Macaluso and Driver, 2001; Bavelier and Neville, 2002; Butler et al., 2012). In addition, several psychophysical studies have reported strong associations between the auditory and motor areas (Jokiniemi et al., 2008; Chen et al., 2009; Yau et al., 2009; Wilson et al., 2010b; Butler et al., 2012). These findings are further supplemented by the neuroimaging studies, reporting shorter pathways between the auditory and motor cortices, especially for multisensory integration (Lang et al., 1990; Zatorre et al., 2007; Foxe, 2009; Keniston et al., 2010; Butler et al., 2012; Chauvigné et al., 2014; Ishikawa et al., 2015). This might explain the strong influence of such audio-tactile cross-modal stimuli in terms of processing temporal (Fujisaki and Nishida, 2009), and certain impact on spatial information (Belardinelli et al., 2009; Jimenez and Jimenez, 2017; for a review see Lu et al., 2013). Nevertheless, despite the vast amount of literature indicating a strong influence of the audio-motor coupling for sensorimotor processing (Ghai et al., 2017c,d,e, 2018), a gap in literature persists concerning its applications in rehabilitation (Danna and Velay, 2017; Ghez et al., 2017), and/or sports (Ghai et al., 2017c).

As mentioned before, proprioception is an integral component of the coordination processes of the body (Gentilucci et al., 1994; Laskowski et al., 2000; Smith et al., 2012; Aman et al., 2014; Ghai et al., 2016, 2017a). Deficits in proprioceptive perception are directly linked with poor sensorimotor and somatosensory functioning (Aman et al., 2014; Ghai et al., 2016), characterized by a wide range of musculoskeletal and neuromuscular disorders (Sacco et al., 1987; Jensen et al., 2002; Ribeiro and Oliveira, 2007; Gay et al., 2010; Konczak et al., 2012; Ghai et al., 2017a). Its predominant role in rehabilitation has been emphasized in several studies (Lephart et al., 1997; Laskowski et al., 2000; Ribeiro and Oliveira, 2007; Rosenkranz et al., 2009; Gay et al., 2010; Aman et al., 2014). Therefore, exploring the possible influences of concurrent auditory feedback on proprioception might provide multifaceted benefits. First and foremost, the outcomes might provide a better understanding of intervention designs in rehabilitation, and sport settings with auditory feedback. Moreover, the evaluation of audio-proprioceptive coupling during an arbitrary action (knee-joint proprioception) might allow a better understanding of trans-modal activity of auditory and motor domains beyond music and language (Altenmüller et al., 2009). Finally, a better comprehensive understanding might be developed to support the psychophysical (Butler et al., 2012), neurophysiological (Ishikawa et al., 2015), studies analyzing the multisensory and cross modal integration between auditory and proprioceptive domains. Till this date, only a handful of researchers have attempted to answer the possible effects of real-time auditory feedback on proprioception (Van Vugt, 2013; Scholz et al., 2016; Danna and Velay, 2017; Dyer J. et al., 2017; Ghez et al., 2017). However, their interpretations on proprioceptive-auditory substitution are mostly speculative. For instance, none of the performed studies excluded vision during the performance of the motor task. As a result, possible influences from the visual modality during multisensory or cross modal integration processes can be expected (Plooy et al., 1998; Verschueren et al., 1998; Lönn et al., 2000). Research indicates the importance of isolating inputs from specific sensorimotor structures to provide a better understanding of direct influence over proprioception (Gay et al., 2010).

In a first attempt we tried to analyse the effects of real-time auditory feedback on clinical aspects of knee joint proprioception in a joint position sense test (Sherrington, 1907; Dover and Powers, 2003; Van Vugt, 2013). Based on interpretations drawn from state feedback control theory (Wolpert and Miall, 1996; Shadmehr and Krakauer, 2008), we expected real-time auditory feedback to cause enhancements in knee-joint proprioception or. Moreover, in a second step, we tried to analyze the effects of subliminal transposition of real-time auditory feedback's frequency on auditory-proprioceptive perceptions. The motivation of this part of study was derived from psychophysical studies revealing strong evidence of convergence between auditory and motor systems for computing frequency (Pantev et al., 2009; Wilson et al., 2009, 2010a), especially within well matched stimuli reflecting a similar event (Foxe, 2009). We expected that if auditory feedback could influence proprioception, understanding the role of frequency in this attained effect could allow a better understanding of the results. We therefore, evaluated influence of any divergent step-wise transposition of frequency with real-time auditory feedback would allow directed modulation of proprioceptive perceptions in terms of knee position.

In this article two experiments are mentioned. The second experiment is an extension of the first study, which was conducted after the analysis of results. The experiment II follows the same design and protocol but differs in terms of the magnitude and direction of step-wise transposition of the frequency of the feedback. se experiments differ based on magnitude and direction of step-wise transposition. We expect the outcomes from this study to provide novel practical implications in rehabilitation and sports settings.

## Methods

### Experiment I

#### Experimental design

This whole CCT was carried out between August 2016 and February 2017. Participants were randomly allocated to experimental or control group. In each group, participants carried out the active (knee-joint) repositioning task with their dominant legs. The experimental group concurrently received real-time and transposed (0.25°/repetition) auditory feedback while performing the active knee re-positioning tasks. The control group received white noise. The experiment consisted of five treatment blocks. Re-positioning tasks without any auditory feedback were performed on the odd numbered blocks. Auditory feedback (real-time, modulated, white noise) was provided in the even treatment blocks. The participants performed 15 repetitions per angle in a block i.e., 30 repetitions per block. The target angle for the repositioning task was 40 and 75°.

#### Participants

Thirty participants, randomly divided in control [8 males/7 females; mean ± SD (age): 23.5 ± 2.5 years], and experimental group I (7 male/8 female; 24.2 ± 3.7 years) volunteered to participate in the study. All participants self-reported as healthy with no history of significant hip, knee, or back injury. Written informed consent was obtained from each participant, and ethical approval was obtained from the Ethics Committee of the Leibniz University Hannover. All participants underwent a baseline test for auditory capabilities (HTTS Audiometry) and were asked to fill a self-reported questionnaire post the experiment. All participants received eight Euros for their participation.

#### Experimental procedure

Participants were comfortably seated with their feet on the floor, their back resting against a wall, and their pelvis stabilized (Tiggelen et al., 2008; Ghai et al., 2016). During the sitting position, the knee joint was maintained at the right angle. This position of the knee joint was considered as 0° and further extension from this position onwards was referred as positive angles from this value (Supplementary File 1). Participants wore wireless headphones (Sennheiser, Wedemark, Germany), and were blindfolded to eliminate visual cues. The experimenter passively moved the dominant leg to a previously identified target position (40 or 75°) in an open kinetic chain and held at the target angle to allow the participant to memorize the position (Selfe et al., 2006; Ghai et al., 2016). The experimenter, a physiotherapist, checked and rechecked the angle while using a handheld goniometer, and motion capture reading to confirm the target angle. The leg was then returned to the initial position, and following a 5 s interval, the participant attempted to reposition the leg at the same joint angle. The participant was instructed to repeatedly re-position the leg to the instructed angle with an instruction “please re-position your leg to the performed angle hold the angle for 2 s and then return it to the starting position.” The experimenter counted 15 repetitions and asked the participants to stop. This protocol was repeated for both the target angles (40 and 75°), across 5 treatment blocks. During the first, third, and fifth treatment blocks no auditory feedback was provided to the participants. However, during the second treatment block the same protocol was followed with real-time auditory feedback i.e., the experimenter initially took the dominant leg to the target angles with real-time auditory feedback. Thereafter, the participants performed the same target angles with real-time auditory feedback. During the fourth block, the experimenter initially positioned the dominant leg passively with real time auditory feedback, after which participants re-positioned their knee unaware of the modulation in frequency of auditory feedback (Supplementary File 2). Dynamic repositioning accuracy was computed to determine discrepancies while consecutively repositioning the knee joint. For instance, the repositioning performance of 40, 38, 43, 37°… the computation of repositioning error was performed by subtracting the performed angle with the previous angle i.e., 38°–40°, 43°–38°, 37°–43°…and so on. After the experiment was concluded, the participants were asked to fill a four-point questionnaire. The questionnaire enquired about the perceived duration of the experiment, the fatigue level, the excerptions perceived if any in the quality of the auditory feedback (for identifying whether participants were consciously able to detect changes in the frequency of the real-time auditory feedback), and subjective rating for compliance with auditory feedback on a 10-point Likert scale. The experimental protocol lasted approximately for 45 min.

#### Real-time auditory feedback mapping

Real-time auditory feedback was generated using Python (version 2.7) and Csound version 6.0. Sound synthesis was based on a band-limited oscillator bank with lowpass filtering. Knee joint angle and angular velocity are mapped onto pitch and amplitude of the auditory feedback, respectively. During sitting the right angle at the knee joint is regarded 0°, and any extension from this point onwards is referred in positive values from this angle. The changes in angles from 0 to 90° of full extension is configured from 120 to 300 Hz of frequency change, respectively. Here, amplitude is a function of square of knee angular velocity which is relevant to kinematic energy. For the amplitude function, exaggerated representation of the angular position was added because, as the frequency increases, human ear gets less sensitive in identifying the same pitch differences. The exaggeration in amplitude can therefore complement the lack of sensitivity, which properly stimulates the human ears. These mapping functions are also provided as a mathematical equation for clarity.

$$\begin{array}{lll} Pit & = & 2\times \theta_{knee.joint}+120\;\left(Hz\right).\\ Amp & = & \;\alpha \omega_{knee.joint}^{2}+\;\beta \left(\cos\left(90^{{}^{\circ}}-\theta_{knee.joint}\right)-k\right). \end{array}$$

In the equations, *Pit* is pitch (audio frequency), θ_*knee*.*joint*_ is the knee joint angle, Amplitude is *Amp*, ω_*knee*.*joint*_ is joint angular velocity. The equation also includes coefficients α, β as well as a constant value, *k*.

Modulation of real-time auditory feedback was subtle and provided in an under-transposition manner. Here, the mapping information between audio frequency and knee angle was manipulated during repetitions. For example, 15 repetitions in a step-down transposition by −0.25° (−0.5 Hz/rep) at the target angle. Frequency was changed per repetition, for instance from 180 to 193 Hz which would be is equivalent to a change of the knee angle from 40 to 36.5° in the constant original mapping (Supplementary Files 3, 6) for 15 repetitions. A sample for both the real-time auditory feedback (Supplementary File 5) and modulated auditory feedback (Supplementary File 6) have been provided.

#### Kinematic analysis

Repositioning error (RE) was assessed in each trial using XSENS MVN Biomech (XSENS Technologies B.V, Netherlands), in a configuration mode limited to the lower body. High reliability and validity of this inertial sensor based motion analysis device has been previously reported (Cooper et al., 2009; Zhang et al., 2013). Seven pre-identified inertial measurement units (IMUs) were placed by a physiotherapist on sacrum, lateral side of femoral shaft, medial surface of tibia, and tarus using velcro straps (Supplementary File 1; Zhao et al., 2016). The angular repositioning data, expressed in sensor coordinate frame was wirelessly recorded with a sampling frequency of 60 Hz in a laptop (Lenovo INC, Hongkong) and saved in MVN file format. Thereafter, the saved file was converted to XML format (MVNX) and imported in a Microsoft Excel spreadsheet. This format incorporates information concerning sensor data, segment kinematics and joint angles. Marked data points (highlighted in MVN file during recording) were matched with MVN recording graphs and the data was manually extracted by two researchers for further calculations. Absolute and constant error were then computed for characterizing the repositioning error in both the magnitude and direction of error, by considering the target angle as the previous consecutive angle to the current performance by the participant.

#### Statistical analysis

Statistical analyses were performed using Statistical Package for Social Science (V. 23.0, SPSS Inc., Chicago, IL). In 2 separate analysis for absolute and constant errors. We analyzed Repositioning Error (the dependent measure), by conducting a Group (Experimental/control) × block (1–5) × Angles (40/75°) RM-ANOVA with repeated measures on the last two factors. Effect sizes of the independent variables were expressed using partial eta squared (η_p_^2^), with effect sizes <0.01 considered to be small, effect sizes between 0.01 and 0.06 considered to be medium and effect sizes >0.14 considered to be large (Sedlmeier and Renkewitz, 2008). *Post-hoc* comparisons were performed using stepwise Bonferroni holm corrections. The overall significance level was set to 5%.

### Results

#### Absolute error

**Figure 1:**
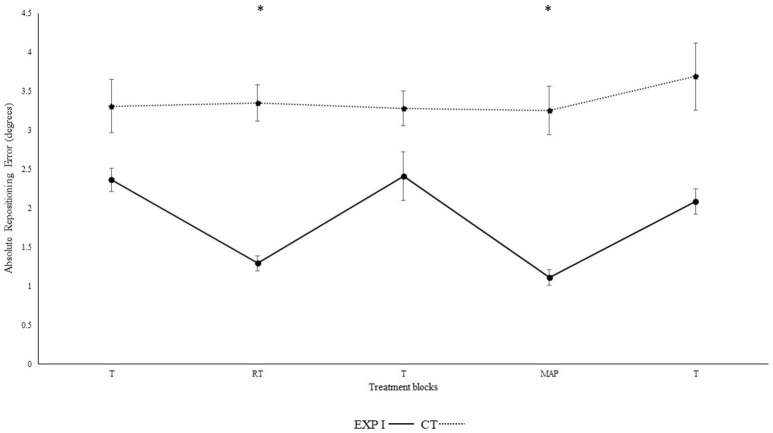
Absolute mean and standard error of repositioning error (°) for the control, experimental group I (Dotted line represents control group. Darkened black line represents experimental group I, T: Proprioceptive test without auditory feedback, RT: Real-time auditory feedback, MAP: Acoustic mapping, CT: Control group, EXP: Experimental group). ^*^Represents significant differences.

Figure 1 illustrates the absolute repositioning accuracy in both groups. The experimental group I, with real-time auditory feedback performed significantly better than the control group without auditory feedback as confirmed by the significant main effect of group [*F*_(1, 28)_ = 6.92, *p* = 0.014, η_p_^2^ = 0.20]. Furthermore, repositioning accuracy depended on block [*F*_(4, 112)_ = 10.16, *p* < 0.001, η_p_^2^ = 0.27]. Differences between block were mainly caused by the auditory feedback in the experimental group I as shown by the interaction block^*^group [*F*_(4, 112)_ = 8.34, *p* < 0.001, η_p_^2^ = 0.23]. A *post-hoc* test confirmed significant differences between the first and second block in the experimental group I (*p* < 0.001), but not in the control group (*p* > 0.999). Furthermore, the second (*p* < 0.001), but not the first (*p* > 0.999) block differed significantly between groups. After the removal of feedback this effect diminished. Accordingly, both groups performed in block 3 not significantly different than in block 1 (experimental group I: *p* > 0.999; control group: *p* > 0.999). Differences between angles were not significant [angles: *F*_(1, 28)_ = 3.39, *p* = 0.076, η_p_^2^ = 0.11; angle^*^group; *F*_(1, 28)_ = 3.65, *p* = 0.066, η_p_^2^ = 0.12; angle^*^block: *F*_(4, 112)_ = 0.46, *p* = 0.714, η_p_^2^ = 0.02; angle^*^block^*^group: *F*_(4, 112)_ = 0.49, *p* = 0.690, η_p_^2^ = 0.02].

#### Constant error

**Figure 2:**
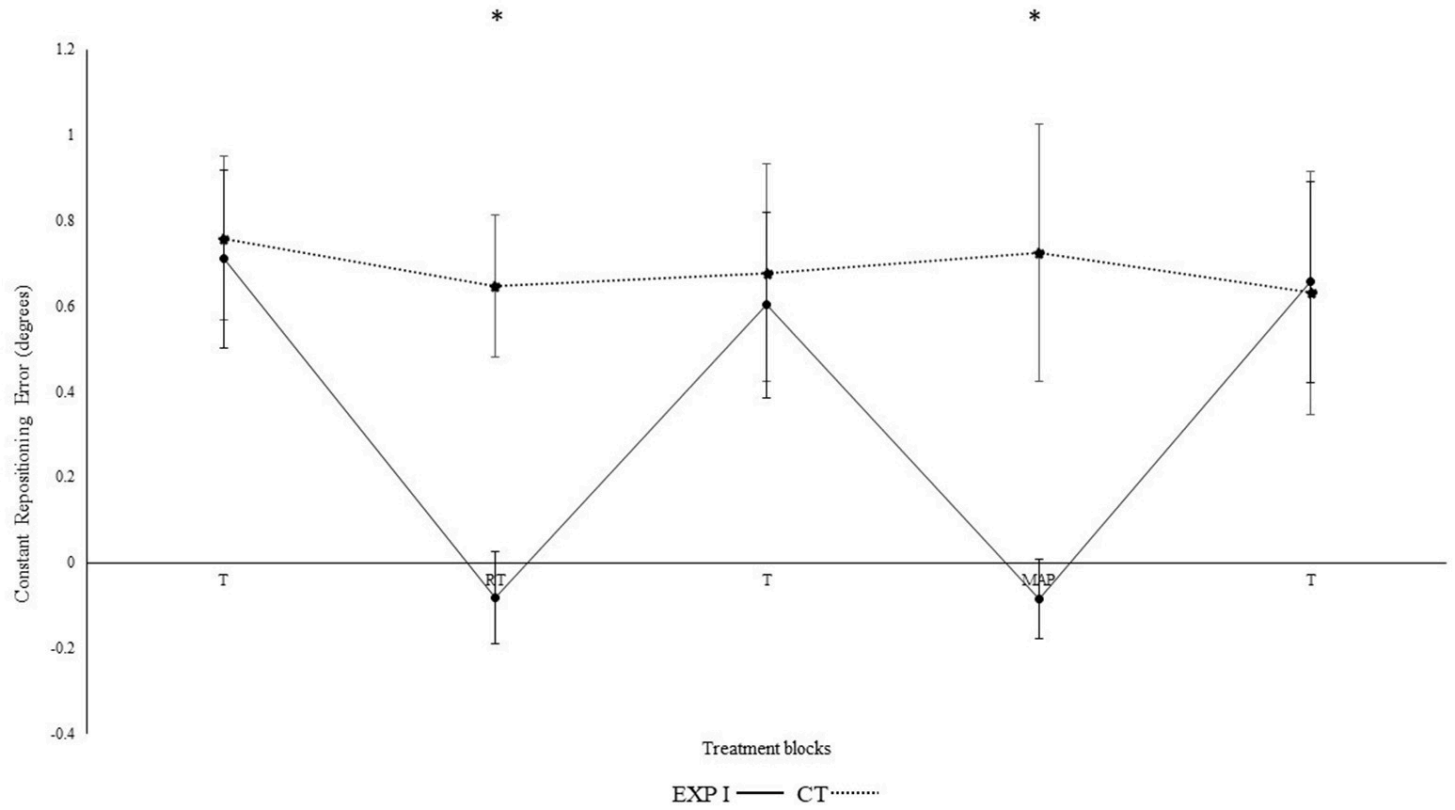
Constant mean and standard error of repositioning error (°) for the control, experimental group I (Dotted line represents control group. Darkened black line represents experimental group I, T: Proprioceptive test without auditory feedback, RT: Real-time auditory feedback, MAP: Acoustic mapping, CT: Control group, EXP: Experimental group). ^*^Represents significant differences.

Figure 2 illustrates the constant repositioning error in both groups. The experimental group I with real-time auditory feedback performed significantly better than the control group without auditory feedback, as confirmed by the significant main effect of group [*F*_(1, 28)_ = 6.150, *p* = 0.019, η_p_^2^ = 0.18]. Furthermore, a main effect was observed for block [*F*_(4, 112)_ = 4.320, *p* = 0.030, η_p_^2^ = 0.13]. Differences between blocks were mainly caused by the auditory feedback in the experimental group I as shown by the interaction block^*^group [*F*_(4, 112)_ = 4.560, *p* = 0.002, η_p_^2^ = 0.140]. A *post-hoc* test confirmed significant differences between the first and second block in the experimental group I (*p* < 0.001), but not in the control group (*p* = 0.360). Furthermore, the second (*p* < 0.001), but not the first (*p* = 0.810) block differed significantly between groups. After the removal of feedback this effect diminished. Accordingly, both groups performed in block 3 not significantly different than in block 1 (experimental group I: *p* > 0.999; control group: *p* > 0.999).

In the 4th block, modulation in frequency of real-time feedback were introduced. We observed significant differences between the 3rd and 4th block of experimental group I (*p* = 0.001), and as compared to the 4th block control group (*p* < 0.001). No such differences were observed between 3rd and 4th block in control group (*p* = 0.660). Likewise, in 5th block both groups performed not significantly different than in 1st and 3rd block (all *p*'s > 0.05). Significant differences were also not evident when the 4th block was compared with the 2nd block (*p* > 0.999) i.e., modulated feedback with un-modulated feedback. Constant error was significantly larger for angle 40° as compared to 75° [*F*_(1, 28)_ = 21.80, *p* < 0.001, η_p_^2^ = 0.44]. However, none of the interactions with the effects of the angles were significant, but not for angle^*^group; [*F*_(1, 28)_ = 0.40, *p* = 0.532, η_p_^2^ = 0.01]; angle^*^block [*F*_(4, 112)_ = 0.36, *p* = 0.838, η_p_^2^ = 0.01] angle^*^block^*^group [*F*_(4, 112)_ = 0.20, *p* = 0.941, η_p_^2^ = 0.01].

### Experiment II

#### Experimental design

This whole trial was carried out between March 2017 and September 2017. Participants were allocated to experimental group II. Due to the identical experimental design as experiment I data from the same control group was utilized for comparison and the data from control group of first experiment was utilized. Here, the participants carried out the active (knee-joint) repositioning task with their dominant legs. The experimental group concurrently received real-time, modulated (±1.3°/repetition) auditory feedback while performing the re-positioning tasks. The control group received white noise. The experiment consisted of five treatment blocks. Re-positioning tasks without any auditory feedback were performed on the odd numbered blocks. Auditory feedback (real-time, modulated, white noise) was provided in the even treatment blocks. The participants performed 15 repetitions per angle in a block i.e., 30 repetitions per block. The target angle for the repositioning task was 40 and 75°.

#### Participants

Twenty healthy participants were included in experimental group II [10 females/10 males; mean ± SD (age): 26.8 ± 3.5 years]. All participants underwent a baseline test for auditory capabilities (HTTS Audiometry). All participants received eight Euros for their participation.

#### Experimental procedure

Same as experiment I.

#### Real-time auditory feedback mapping

Real-time auditory feedback was generated using Python (version 2.7) and Csound version 6.0. Sound synthesis was based on a band-limited oscillator bank with lowpass filtering. Knee joint angle and angular velocity are mapped onto pitch and amplitude of the auditory feedback, respectively. During sitting the right angle at the knee joint is regarded 0°, and any extension from this point onwards is referred in positive values from this angle. The changes in angles from 0 to 90° of full extension is configured from 120 to 300 Hz of frequency change, respectively. Here, amplitude is a function of square of knee angular velocity which is relevant to kinematic energy.

The modulation of real-time auditory feedback was subtle and provided in an over/under-transposition manner. Here as well, the frequency of the auditory feedback was manipulated per repetition, for 15 repetitions. However, the gradient of change was larger i.e., ±2.6 Hz (equivalent to ±1.3° change). Here during step down-up the change in frequency was equivalent as a change from 180 Hz (40°) to 167 Hz (34.8°) in the 5th repetition, and then to 182.6 Hz (41.7°) for the 10th repetition, and finally to 167 Hz (34.8°) for the 15th repetition. For instance, in step up-down manner 15 repetitions were accounted in three continuous steps: first five repetitions i.e., 1–5 transposition were performed in step-up manner i.e., 40, 41.3, 42.6, 43.9, 45.2°. Thereafter, for repetitions 6–10 continuously the direction of transposition was changed in step-down manner i.e., 43.9, 42.6, 41.3, 40, 38.7°. Lastly, for the final 11–15 repetitions the transposition was again changed to step-up manner i.e., 40, 41.3, 42.6, 43.9, 45.2°. This transposition change was randomized with step down-up approach during the study. For better clarity see Supplementary Files 4, 7.

The application of transposition was counterbalanced across four sub-groups i.e., sub-group I (40°: under-over-under, 75°: over-under-over), sub-group II (40°: over-under-over, 75°: over-under-over), sub-group III (40°: over-under-over, 75°: under-over-under), and sub-group IV (40°: under-over-under, 75°: under-over-under). Therefore, the number of participants was balanced across the conditions and increased to 20 i.e., 5 in each sub-group. A sample for both the real-time and modulated auditory feedback (Supplementary Files 6, 7) have been provided.

#### Kinematic analysis

Same as experiment I.

#### Statistical analysis

Like experiment I, in 2 separate analysis absolute and constant errors were compared with control group. Here, the control group from experiment I was utilized. We analyzed Repositioning Error (the dependent measure), by conducting a Group (Experimental/control) × blocks (1-5) × Angles (40/75°) RM-ANOVA with repeated measures on the last two factors. Additionally, data were decomposed for the 4th block, where the frequency was modulated, across four different sub-groups. Here, the data were normalized on an individual level to the real-time non-modulated auditory feedback by subtraction. The four sub-groups differed in performance of episodes of transposition i.e., sub-group I (40°: under-over-under, 75°: over-under-over), sub-group II (40°: over-under-over, 75°: over-under-over), sub-group III (40°: over-under-over, 75°: under-over-under), and sub-group IV (40°: under-over-under, 75°: under-over-under). Here, each episode represented the mean of five subsequent movements. For the analysis the values for the over-transposition were inverted. Here, analysis of variance was performed on normalized repositioning errors as dependent variable and between subject factor sub-groups (I, II, III, IV) and within subject factor episodes (1–3) and angles (40/75°). Here, each episode represented the mean of five subsequent movements. *Post-hoc* comparisons were performed using step wise Bonferroni holm corrections.

### Results

#### Absolute error

**Figure 3:**
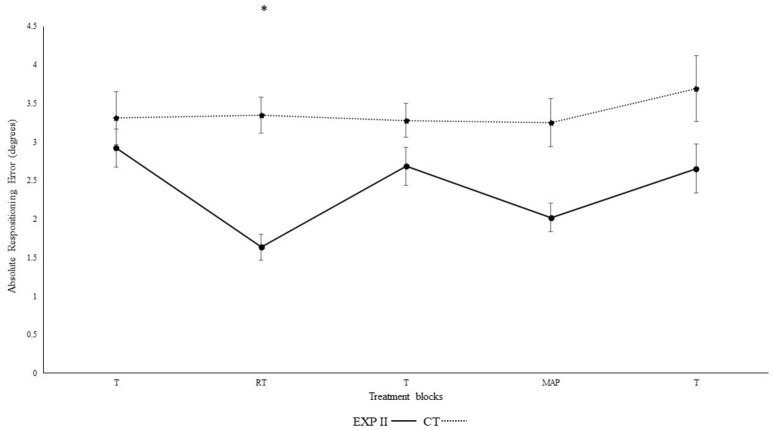
Absolute mean and standard error of repositioning error (°) for the control and experimental group II (Dotted line represents control group. Darkened black line represents experimental group II, T: Proprioceptive test without auditory feedback, RT: Real-time auditory feedback, MAP: Acoustic mapping, CT: Control group, EXP: Experimental group). ^*^Represents significant differences.

Figure 3 illustrates the absolute repositioning error in both groups. Significant differences were observed in between blocks [*F*_(4, 132)_ = 38.3, *p* < 0.001, η_p_^2^ = 0.54] and interaction was evident for block^*^group [*F*_(4, 132)_ = 4.4, *p* < 0.01, η_p_^2^ = 0.12]. A *post-hoc* test confirmed significant differences between the first and second block in the experimental group I (*p* < 0.001), but not in the control group (*p* = 0.940). Furthermore, the second (*p* < 0.001), but not the first (*p* = 0.30) block differed significantly between groups. After the removal of feedback this effect diminished. Accordingly, both groups performed in block 3 not significantly different than in block 1 (experimental group I: *p* > 0.999; control group: *p* > 0.999). None of the other results were significant group [*F*_(1, 33)_ = 2.0, *p* = 0.15, η_p_^2^ = 0.06], angles [*F*_(1, 33)_ > 0.01, *p* = 0.970, η_p_^2^ < 0.001], angle^*^group [*F*_(1, 33)_ = 0.01, *p* = 0.920, η_p_^2^ < 0.001], angle^*^block [*F*_(4, 132)_ = 0.3, *p* = 0.780, η_p_^2^ = 0.01], angle^*^block^*^group [*F*_(4, 132)_ = 0.77, *p* = 0.490, η_p_^2^ = 0.02].

#### Constant error

**Figure 4:**
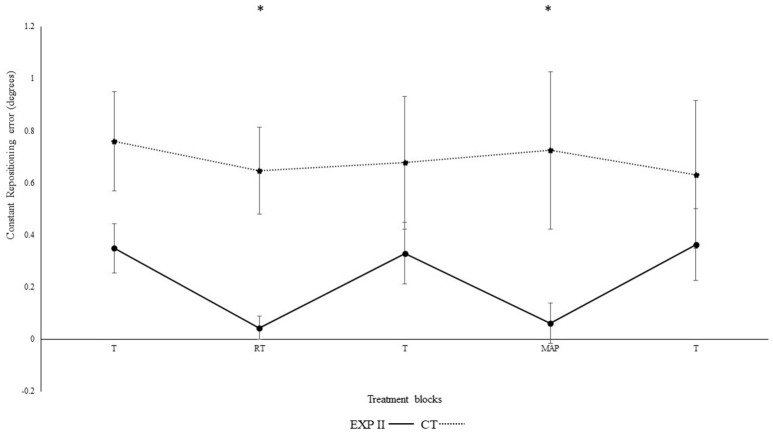
Constant mean and standard error of repositioning error (°) for the control, and experimental group II. (Dotted line represents control group. Darkened black line represents experimental group I, gray line represents experimental group II, T: Proprioceptive test without auditory feedback, RT: Real-time auditory feedback MAP: Acoustic mapping step-down 0.25/rep for exp I, 1.3/rep for exp II, CT: Control group, EXP: Experimental group). ^*^Represents significant differences.

Figure 4 illustrates the constant repositioning accuracy in both groups. The repositioning accuracy depended on block [*F*_(4, 132)_ = 14.2, *p* < 0.001, η_p_^2^ = 0.3]. Differences between conditions were mainly caused by the auditory feedback in the experimental group I as shown by the interaction block^*^group [*F*_(4, 112)_ = 4.56, *p* = 0.002, η_p_^2^ = 0.14]. A *post-hoc* test confirmed significant differences between the first and second block in the experimental group I (*p* = 0.003), but not in the control group (*p* = 0.730). Furthermore, the second (*p* = 0.001), but not the first (*p* > 0.999) block differed significantly between groups. After the removal of feedback this effect diminished. Accordingly, both groups performed in block 3 not significantly different than in block 1 (experimental group I: *p* > 0.999; control group: *p* > 0.999). In the fourth block, subliminal modulation in frequency of real-time feedback were introduced. We observed no significant differences in the 4th block of experimental group II (*p* = 0.220), control group (*p* = 0.770) as compared to the 3rd block. This difference was however, significant when compared to the control group (*p* = 0.010). Likewise, both groups performance in 5th block did not significantly different than in block 1, and 3 (experimental group II: *p* > 0.999; control group: *p* > 0.999). Significant differences were not evident when modulated feedback in 4th block was compared with un-modulated feedback in the 2nd block (*p* > 0.999). Differences were significant in between the angles [*F*_(1, 33)_ = 19.6, *p* < 0.01, η_p_^2^ = 0.37] i.e., constant errors were larger for 40° as compared to 75° and for angle^*^group; [*F*_(1, 33)_ = 14.5, *p* = 0.001, η_p_^2^ = 0.31], but not for group [*F*_(1, 33)_ < 0.01, *p* = 0.990, η_p_^2^ < 0.01], angle^*^block [*F*_(4, 132)_ = 0.6, *p* = 0.650, η_p_^2^ = 0.02], angle^*^block^*^group [*F*_(4, 132)_ = 0.89, *p* = 0.470, η_p_^2^ = 0.03].

### Transposition condition

**Figure 5:**
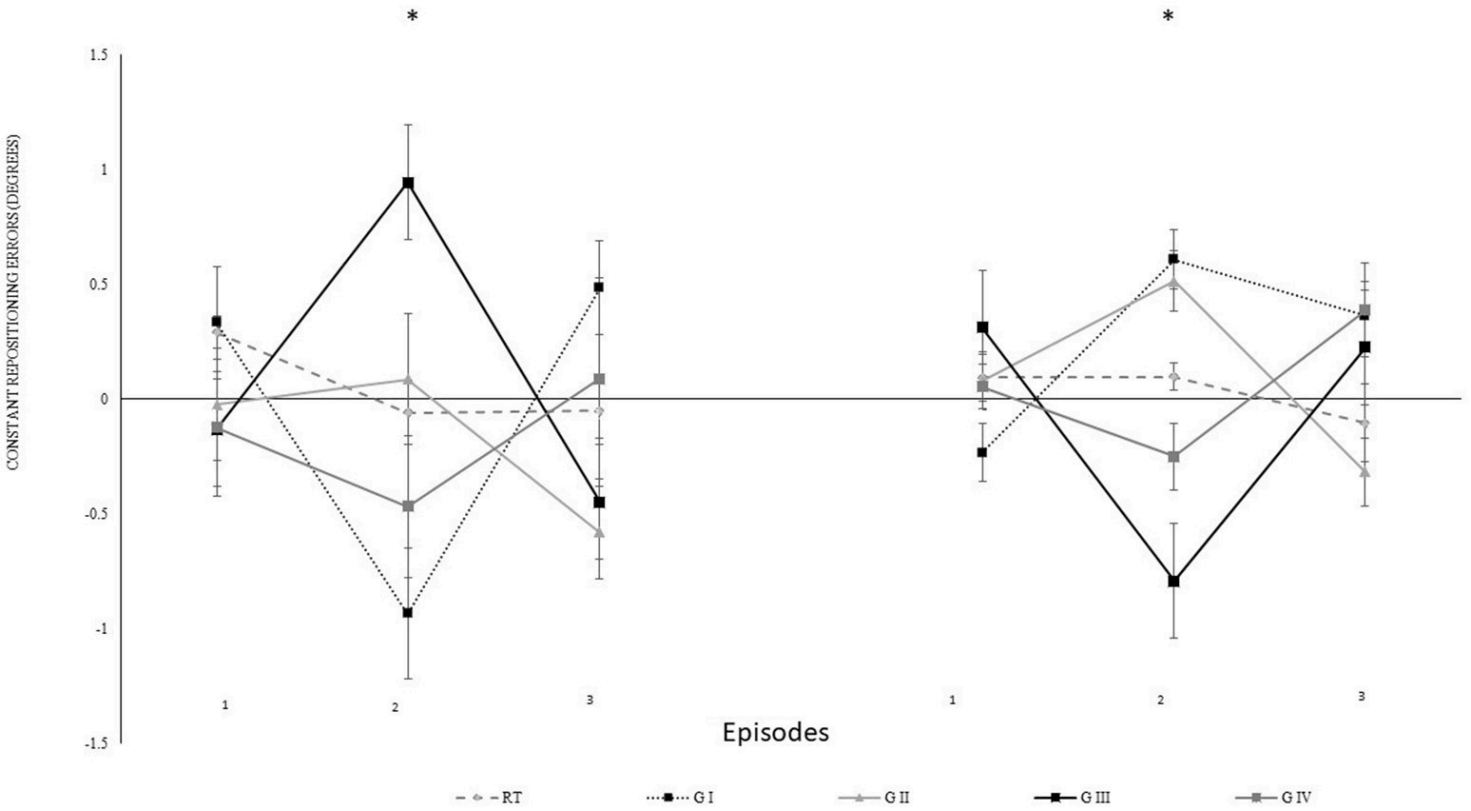
Constant mean and standard error of repositioning error (°) for the experimental II, 2nd and 4th block, also for episodes (1–3). Difference in proprioceptive perceptions in between decomposed mapping conditions have been described for 5 sub-groups i.e., RT: real-time auditory feedback, sub-group I (G I: 40°: under-over-under, 75°: over-under-over), sub-group II (G II: 40°: over-under-over, 75°: over-under-over), sub-group III (G III:40°: over-under-over, 75°: under-over-under), and sub-group IV (G IV: 40°: under-over-under, 75°: under-over-under), and across 3 treatment blocks. The values on left represent 40°, and right 75° (RT: Real-time kinematic auditory feedback). ^*^Represents significant differences.

For specifying the effect of transposition, we decomposed the data from the 4th block. We computed constant errors separately for every five repetitions with transposition in the same directions. Each episode began with either over-under-over or under-over-under transposition. Figure 5 shows the constant errors separately for participants with different episodes. Here, four sub-groups were distinguished with five participants each i.e., sub-group I performed for (40°: under-over-under, 75°: over-under-over), sub-group II (40°: over-under-over, 75°: over-under-over), sub-group III (40°: over-under-over, 75°: under-over-under), and sub-group IV (40°: under-over-under, 75°: under-over-under). Figure 5 indicates that the re-positioning performance tended to compensate in the opposite direction in which the auditory feedback was manipulatively directed i.e., the participants knee flexion when the feedback was over transposed and vice versa for the under transposition. For the analysis, the over transposition repositioning errors were multiplied with −1.

The data were normalized for the analysis according to individual real-time auditory feedback performance of each participants. Further, step-up transposition findings were multiplied with −1 to allow the direction of transposition to be similar for all episodes (1–3). The statistical analysis revealed that episodes had no significant effect [Episode: *F*_(3.16)_ = 1.51, *p* = 0.414, η_p_^2^ = 0.16; angle^*^episode: *F*_(3.16)_ = 0.72, *p* = 0.556, η_p_^2^ = 0.12; block^*^episode: *F*_(6.32)_ = 1.43, *p* = 0.233, η_p_^2^ = 0.22; angle^*^episode^*^group: *F*_(6.32)_ = 1.04, *p* = 0.420, η_p_^2^ = 0.16] indicating that over- and under-transpositions did not differ in their impact. However, the transpositions were more effective in the second compared to the first episode (*p* = 0.002) as confirmed by post-hoc comparisons to the main effect of episode [*F*_(2, 32)_ = 7.39, *p* = 0.002, η_p_^2^ = 0.32]. Differences between the first and the third (*p* = 0.267) or the second and the third episode (*p* = 0.090) were not significant.

To scrutinize whether the altered mapping between auditory feedback and angle changed the repositioning error we performed *t*-tests against zero separately for episodes (1–3). The results confirmed significant differences to zero in episode 2 (*p* < 0.001) and episode 3 (*p* = 0.029) but not block 1 (*p* = 0.208).

## Discussion

Results from the current experiment demonstrate beneficial effects of real-time auditory feedback on knee re-positioning accuracy. Significant enhancement in re-positioning accuracy was observed for both absolute (*p* < 0.001) and constant error (*p* < 0.01) and both within and across the experimental I and II (For clarity see Figures 1–4), with real-time auditory feedback. These findings agree with previous literature indicating strong associations between the auditory and motor domains (Foxe, 2009; Butler et al., 2012; Schmitz et al., 2013; Ishikawa et al., 2015), and support the possibility of the auditory-proprioceptive substitution hypothesis raised by Altenmüller et al. (2009), Danna and Velay (2017), and Scholz et al. (2015). In this experiment, the enhancement in re-positioning accuracy with real-time auditory feedback could possibly be associated with the “guidance hypothesis” (Schmidt, 1991; Park et al., 2000). The auditory feedback could have made it easier for the participant to identify the target angles, reduce errors, and re-produce the instructed target angles more precisely. This enhancement in re-producibility of target angles could also be due to high spatio-temporal precision of combined audio-motor domains (Hancock et al., 2013; van Vugt and Tillmann, 2015; Dyer J. et al., 2017), which also might have lowered the somatosensory mismatch negativity (Butler et al., 2012). These changes were also affirmed by Fujioka et al. (2012a). The authors reported modulations in the functional reorganization of spatio-temporal patterns of neuromagnetic β activity (between auditory and sensorimotor modalities; Fujioka et al., 2012a,b). Moreover, the enhanced activation in multisensory integration sites (such as neocortex, superior colliculi, striatum, and cerebellum) and action observation system (Superior temporal sulcus, BA 44, 45) might have aided in enhancing the saliency of executed movement patterns (Schmitz et al., 2013; Stein et al., 2014; Chabrol et al., 2015).

These enhancements in re-positioning accuracy however, were not as stable. Once the auditory feedback was removed in the third treatment block, the re-positioning errors returned to their initial levels. This lack of retention in re-positioning accuracy might be linked with over dependency of the participants with the concurrent feedback (Schmidt, 1991). Park et al. (2000) reported that the concurrent feedback can make the learners dependent on the feedback for maintaining their performances, possibly by bypassing the important internal correction and/or error detecting mechanisms (Schmidt, 1991). Moreover, the concurrent feedback might also limit a performer's initial movement error's (Winstein and Schmidt, 1990), which are thought to represent internal variability of the motor system and are considered as essential for the learning process (see dynamic system theory; Clark and Phillips, 1993). Similarly, the rapid change in knee re-positioning accuracy with substitution of auditory feedback could be affirmed with changes in attentional resources. Recently, Ghai et al. (2016) demonstrated that proprioception is adversely impacted under the influence of higher information processing constrains. However, Hopkins et al. (2017) suggested that cross modal cueing can avoid information overload in the native sensory modality by directing task-irrelevant information toward the underused sensory modality (Hameed et al., 2009). Here as well, the introduction of auditory feedback could have possibly allowed enhancements in re-positioning accuracy by transferring excess information in the sister domain (Lohnes and Earhart, 2011; Ghai et al., 2017b).

**Figure 6:**
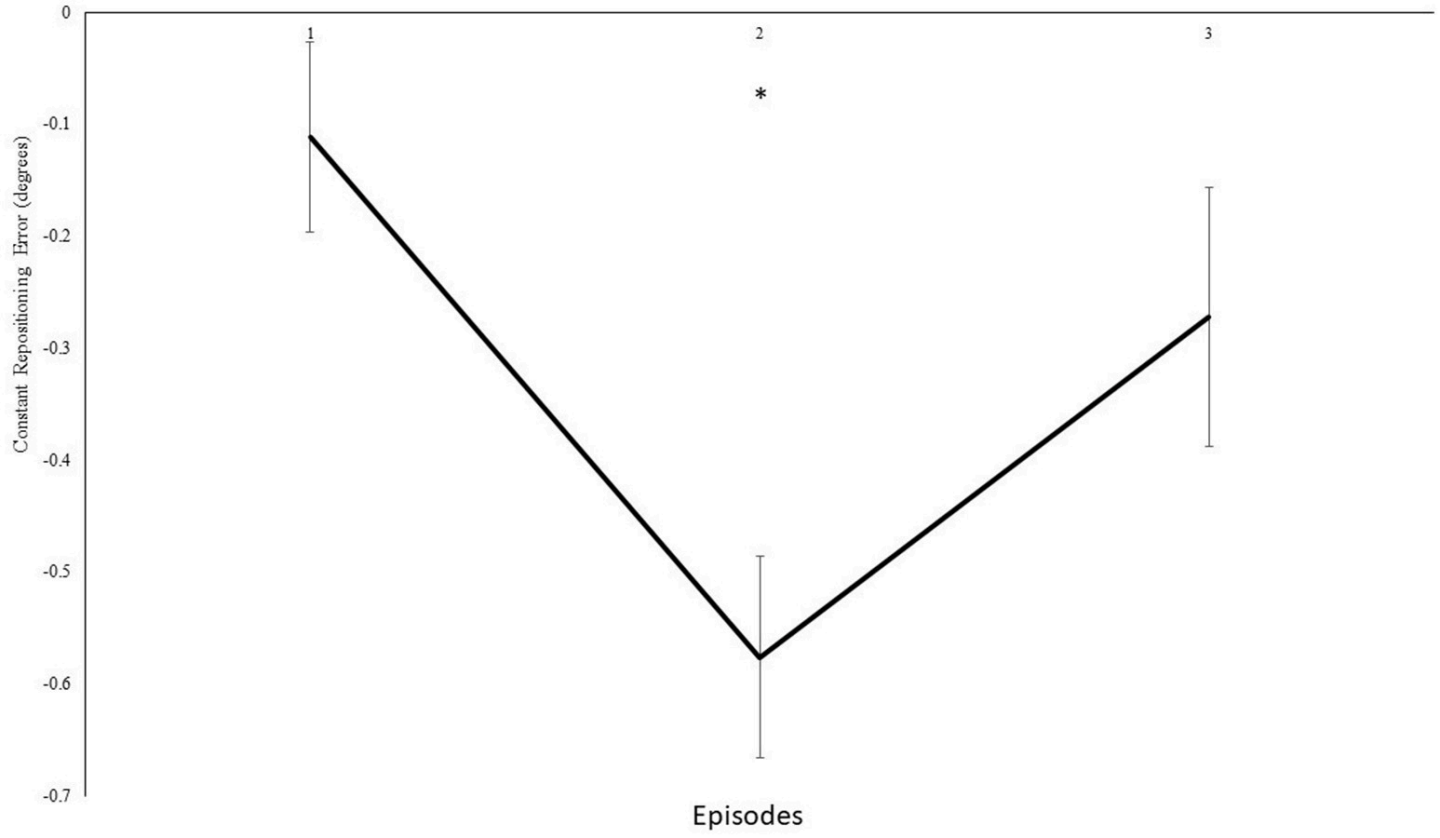
Constant mean and standard error of the repositioning error (°) for the experimental II, 4th block, the values of transposition are normalized, and step-up transpositions have been multiplied with −1 to allow the direction of transposition to be similar for all three blocks. Mean values across the 2 angles for episodes (1–3). ^*^Represents significant differences.

Furthermore, we analyzed modulations in knee repositioning performance with modulations in frequency of the auditory feedback. We confirmed with a self-reported questionnaire that participants were not able to consciously perceive any differences introduced in the frequency of the auditory feedback in both group I and II. However, our results demonstrate that these modulations were dependent on the magnitude of modulation introduced in the frequency. In experiment group I, the step-wise modulations were produced in a step-down transposition by 0.5 Hz/repetition (0.25° or 0.2%/rep). Although a trend toward step-wise modulation was observed for some individual participants, possibly due to their different inherent auditory perceptual capabilities (Kagerer et al., 2014), these differences could not be proven statistically (*p* > 0.05), when compared with real-time auditory feedback condition. Thereafter, upon deliberate examination in multiple pilot trials, a step-wise modulation by 2.6 Hz/repetition (1.3° or 1.1%/rep) was identified and included. The step-wise modulation was performed in three steps, across both the directions i.e., under, over, under transposition across 15 repetitions and vice versa. The direction was changed after five repetitions to avoid conscious perceptions i.e., five repetitions accounted for 6.5° change in one direction, and 19.5° overall change 15 repetitions. On the contrary, in experiment I only 3.5° change was evitable across 15 repetitions. During the initial analysis, no significant differences in knee repositioning accuracy were observed, possibly due to the negation of directional errors in perceptions across the blocks by step-up/down transposition. Therefore, upon factorial re-analysis of decomposed data for directional changes for knee repositioning, we observed significant effect of modulated auditory feedback as compared to real-time auditory feedback. The participants tried to compensate their knee re-positioning by tending to either extend or flex their knee's more with step-down and step-up transposition in frequency (Figure 5), respectively. In our analysis we observed a significant effect of transposition as compared to real-time auditory feedback and demonstrated a combined effect of the transposition to manipulate knee repositioning. As demonstrated in Figure 6, the participants could have taken time to adjust their re-positioning according to the dynamically transposed auditory feedback, or the significance in the next two episodes might be due to practice effect. Previously, published literature has demonstrated the effectiveness of audio-motor coupling due to subliminal changes in rhythmic auditory feedback (Repp, 2000, 2001; Tecchio et al., 2000; Kagerer et al., 2014). These findings also build up on psychophysical studies demonstrating the cross-sensory impacts of frequency modulation between auditory and motor domains (Foxe, 2009; Butler et al., 2012). We demonstrate that subliminal modulation of frequency can lead to goal-directed changes in knee repositioning. To the best of our knowledge, this study for the first time demonstrates modulation in knee repositioning due to subliminal changes in frequency of real-time auditory feedback. Previously, published literature has only demonstrated this association of audio-motor coupling with subliminal changes in inter stimulus interval for rhythmic auditory feedback (Repp, 2000, 2001; Tecchio et al., 2000; Kagerer et al., 2014).

Finally, building upon the strong correlation suggested for proprioceptive, re-positioning tasks (Vidoni and Boyd, 2009; Van Vugt, 2013), and similar open kinetic chain training regimes in rehabilitation (Tagesson et al., 2008; Fukuda et al., 2013; see review Glass et al., 2010), we believe enhancements observed in this experiment can have a range of practical implications in both rehabilitation and sports settings. Fukuda et al. (2013), for instance reported considerable enhancement in quadriceps, hamstrings strength recovery in patients with ACL reconstruction while performing similar non-weight bearing open kinetic chain movements at the knee joint. Moreover, changes in movement patterns associated with subliminal changes in frequency can also have practical implications. For instance, enhancement in breathing (Murgia et al., 2016), music learning (Hol, 2011; Lahav et al., 2013), arm reaching (Maulucci and Eckhouse, 2001; Schmitz et al., 2014; Scholz et al., 2016), gait (Maulucci and Eckhouse, 2011; Zhang et al., 2013; Mezzarobba et al., 2017), sports (Eriksson and Bresin, 2010; Sigrist et al., 2013), performance with real-time auditory feedback has been demonstrated in a few studies. Here, subliminal modulation in frequency during training can be introduced to enhance variability in movement patterns, which further can lead to a dynamic learning pattern (Stein et al., 2014). Moreover, introduction of subliminal changes can be used to prompt the patient or sports person to exceed their performance parameters without consciously perceiving them i.e., possibly reducing movement re-investment (see Masters and Maxwell, 2008). Future studies can evaluate these aspects of modulation in training paradigms in both sports and rehabilitation settings. Finally, the subjective rating of the compliance of auditory feedback in the experiment revealed higher rating for the auditory feedback (6.1 ± 1.0) as compared to the control condition (3.5 ± 1.5). A higher compliance with auditory feedback in past has been associated with enhanced motivation, attention and arousal (Menon and Levitin, 2005; Cha et al., 2014). Thereby, possibly supporting the applications of such type of concurrent auditory feedback in rehabilitation settings.

## Author contributions

AE, GS, and SG developed the research question; SG, AE, and GS developed the research paradigm; SG conducted the experiment, collected the data, and wrote main parts of the paper; GS performed the statistical analysis supported by AE; SG contributed to the results section; T-HH was responsible for technical implementing and customization of the sonification system; AE supervised the project. All authors critically revised the paper.

### Conflict of interest statement

The authors declare that the research was conducted in the absence of any commercial or financial relationships that could be construed as a potential conflict of interest.
