# Supplementary material for: Auditory Proprioceptive Integration: Effects of Real-Time Kinematic Auditory Feedback on Knee Proprioception
Source: Front Neurosci. 2018 Mar 8;12:142. doi: 10.3389/fnins.2018.00142 (PMC5852112; doi:10.3389/fnins.2018.00142)
Supplement: Supplementary file 1 [file Presentation1.PPTX]

## Slide 1
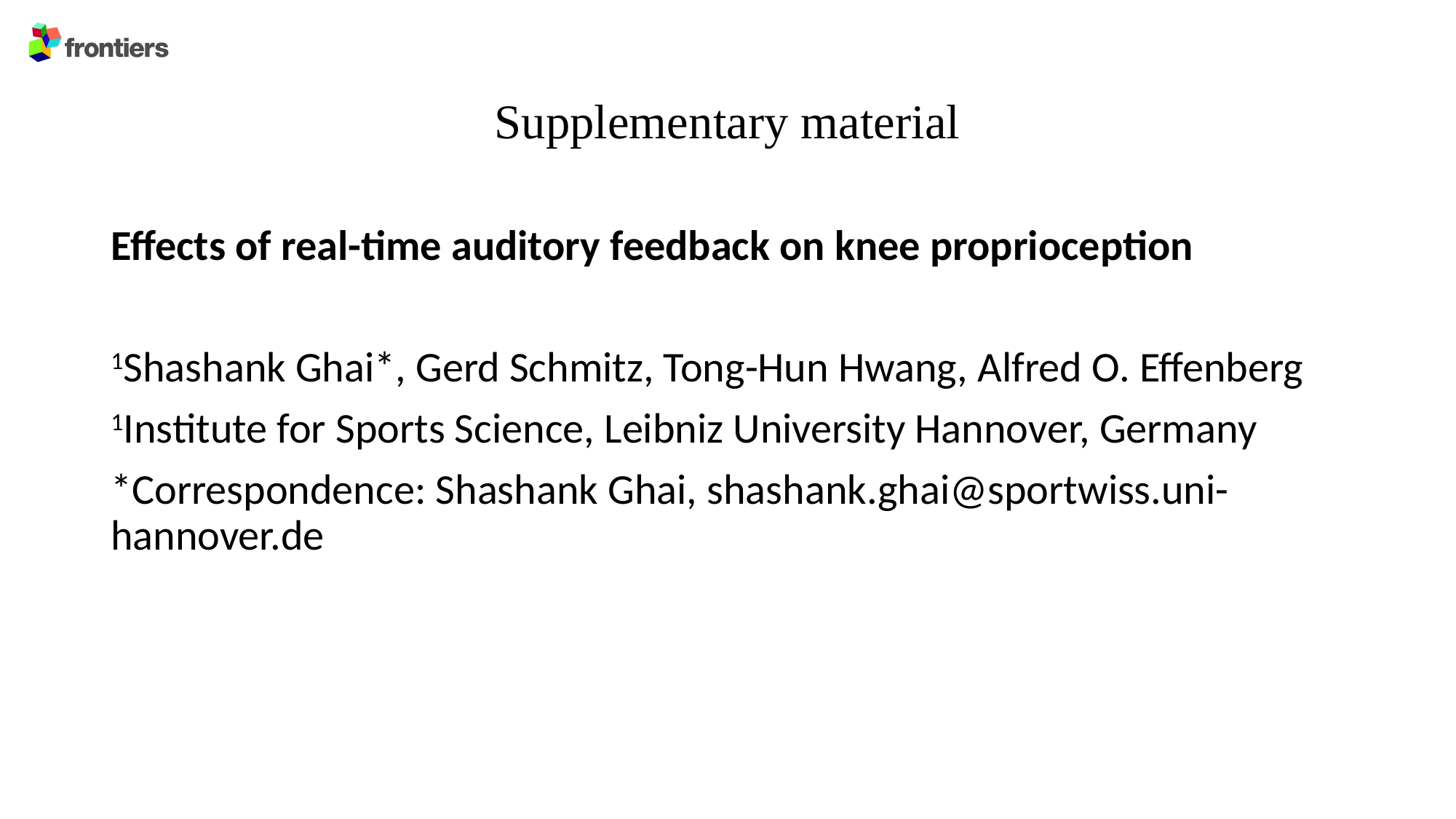

# Supplementary material
Effects of real-time auditory feedback on knee proprioception
1Shashank Ghai*, Gerd Schmitz, Tong-Hun Hwang, Alfred O. Effenberg
1Institute for Sports Science, Leibniz University Hannover, Germany
*Correspondence: Shashank Ghai, shashank.ghai@sportwiss.uni-hannover.de

## Slide 2
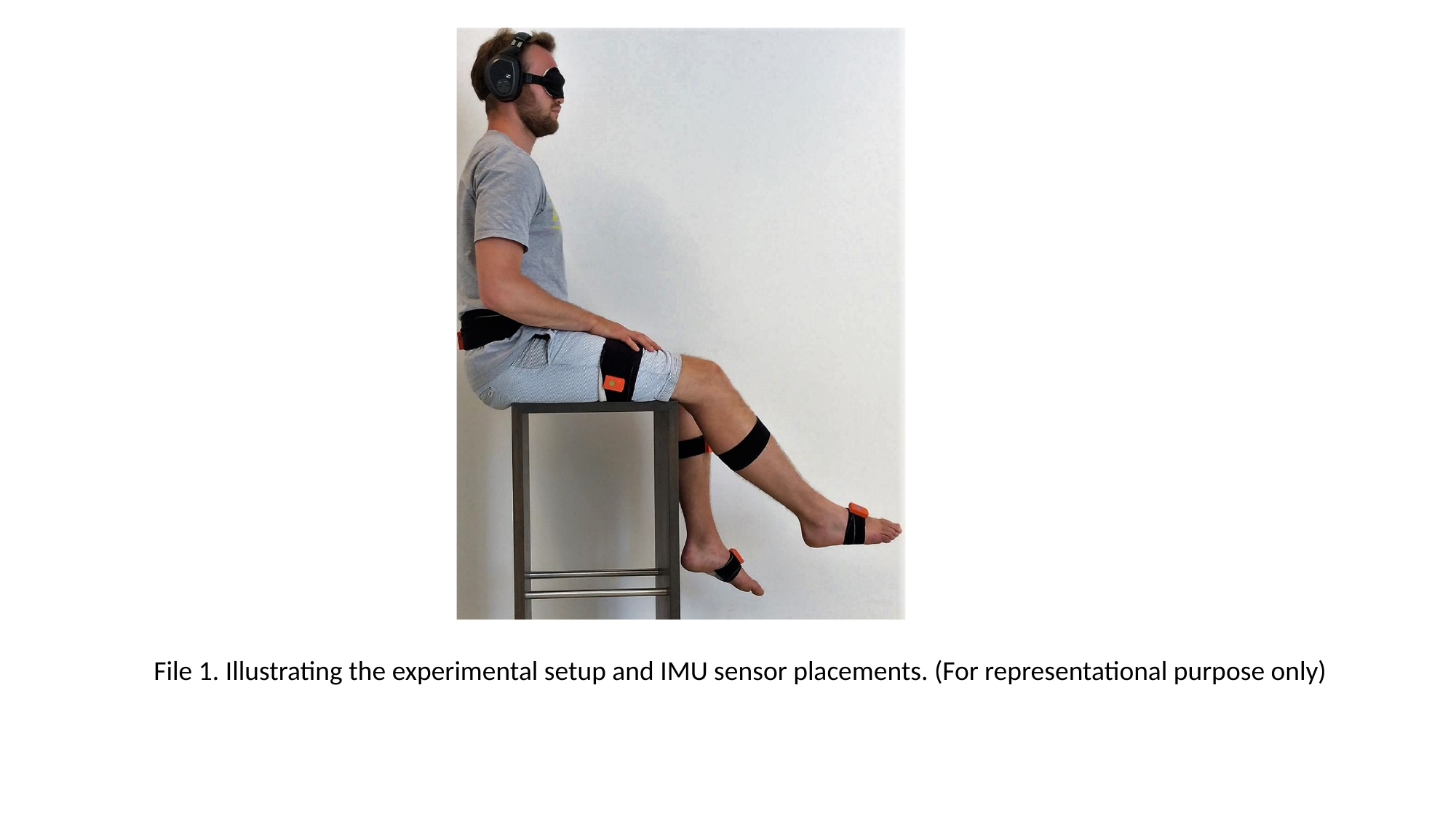

File 1. Illustrating the experimental setup and IMU sensor placements. (For representational purpose only)

## Slide 3
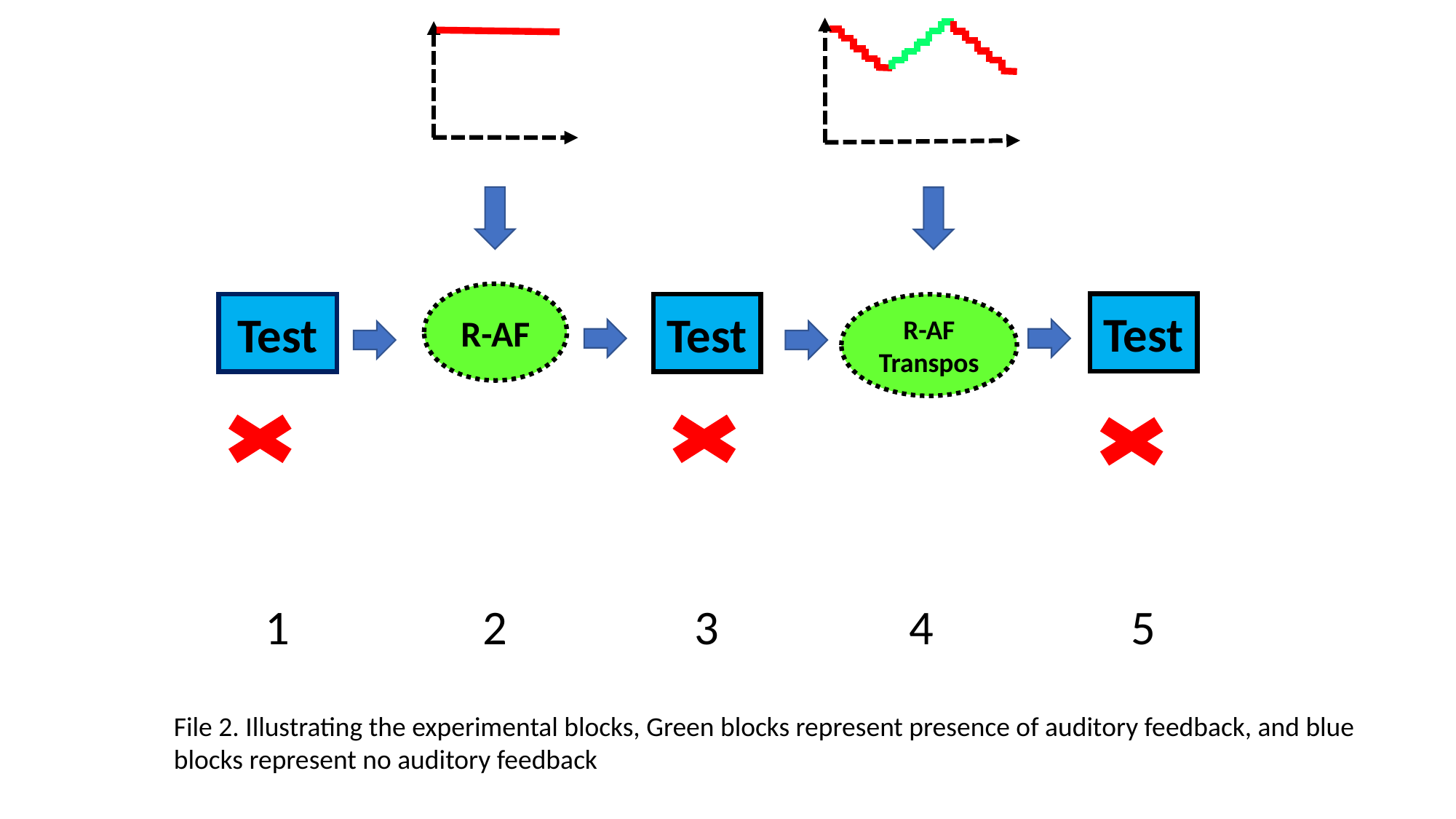

40
387
38.7
37.4
37.4
36.1
36.1
34.8
R-AF
Test
Test
Test
R-AF Transpos
5
4
3
2
1
File 2. Illustrating the experimental blocks, Green blocks represent presence of auditory feedback, and blue blocks represent no auditory feedback

## Slide 4
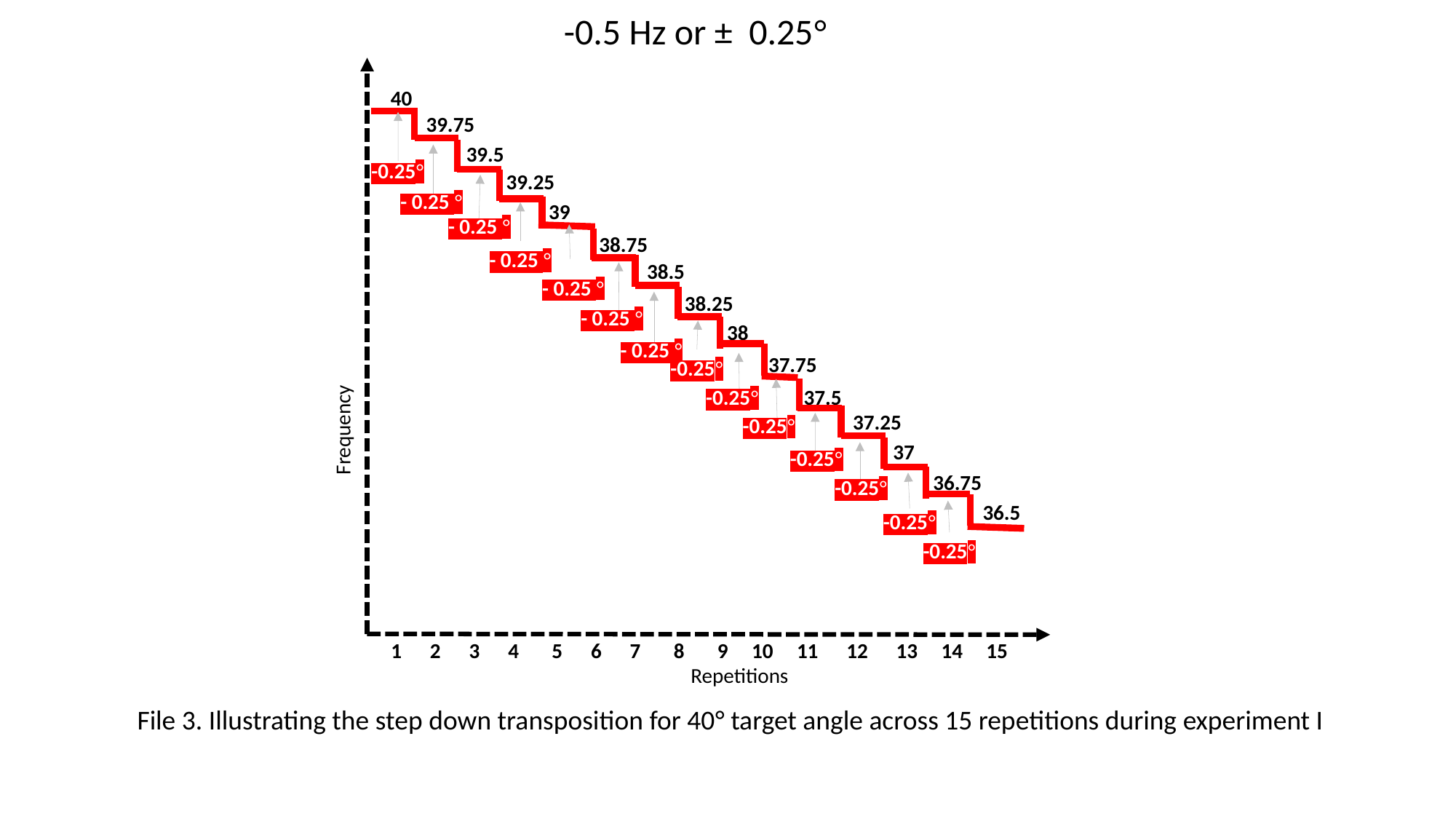

-0.5 Hz or ± 0.25°
40
39.75
39.5
-0.25°
39.25
- 0.25 °
39
- 0.25 °
38.75
- 0.25 °
38.5
- 0.25 °
38.25
- 0.25 °
38
- 0.25 °
37.75
-0.25°
37.5
-0.25°
37.25
-0.25°
Frequency
37
-0.25°
36.75
-0.25°
36.5
-0.25°
-0.25°
1 2 3 4 5 6 7 8 9 10 11 12 13 14 15
Repetitions
File 3. Illustrating the step down transposition for 40° target angle across 15 repetitions during experiment I

## Slide 5
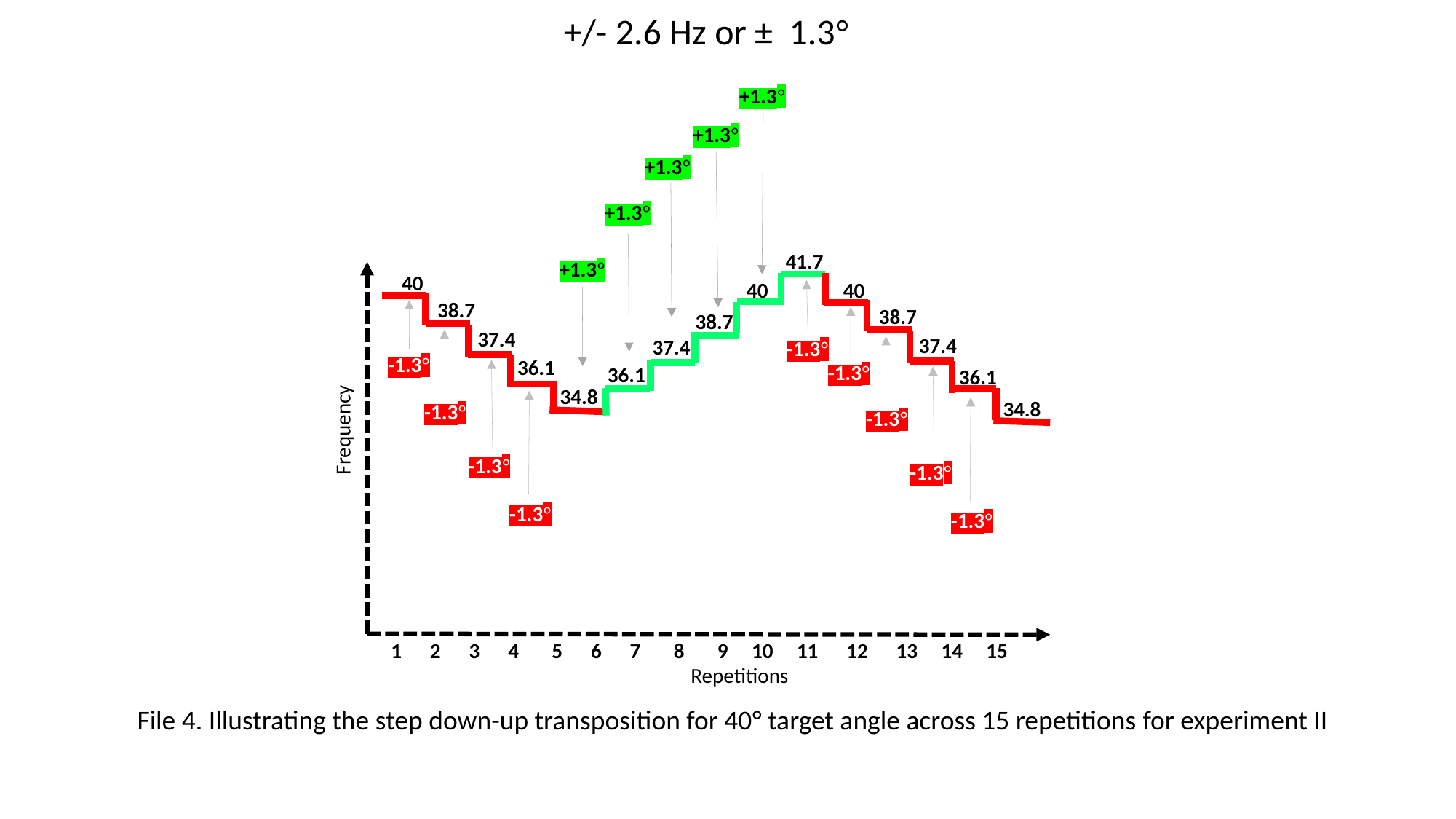

+/- 2.6 Hz or ± 1.3°
+1.3°
+1.3°
+1.3°
+1.3°
41.7
+1.3°
40
40
40
38.7
38.7
38.7
37.4
37.4
37.4
-1.3°
-1.3°
36.1
-1.3°
36.1
36.1
34.8
34.8
-1.3°
-1.3°
Frequency
-1.3°
-1.3°
-1.3°
-1.3°
1 2 3 4 5 6 7 8 9 10 11 12 13 14 15
Repetitions
File 4. Illustrating the step down-up transposition for 40° target angle across 15 repetitions for experiment II

## Slide 6
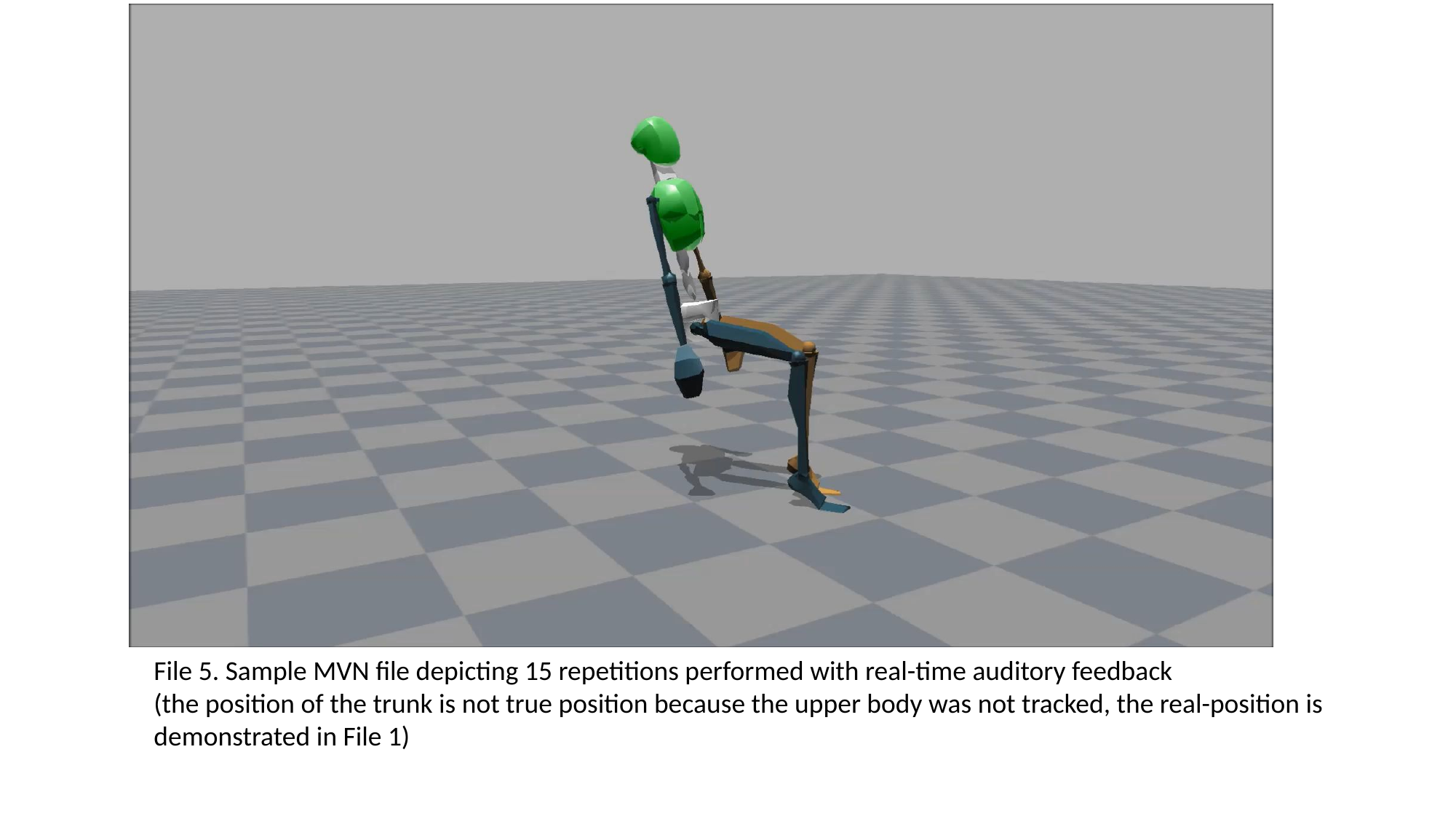

File 5. Sample MVN file depicting 15 repetitions performed with real-time auditory feedback
(the position of the trunk is not true position because the upper body was not tracked, the real-position is demonstrated in File 1)

## Slide 7
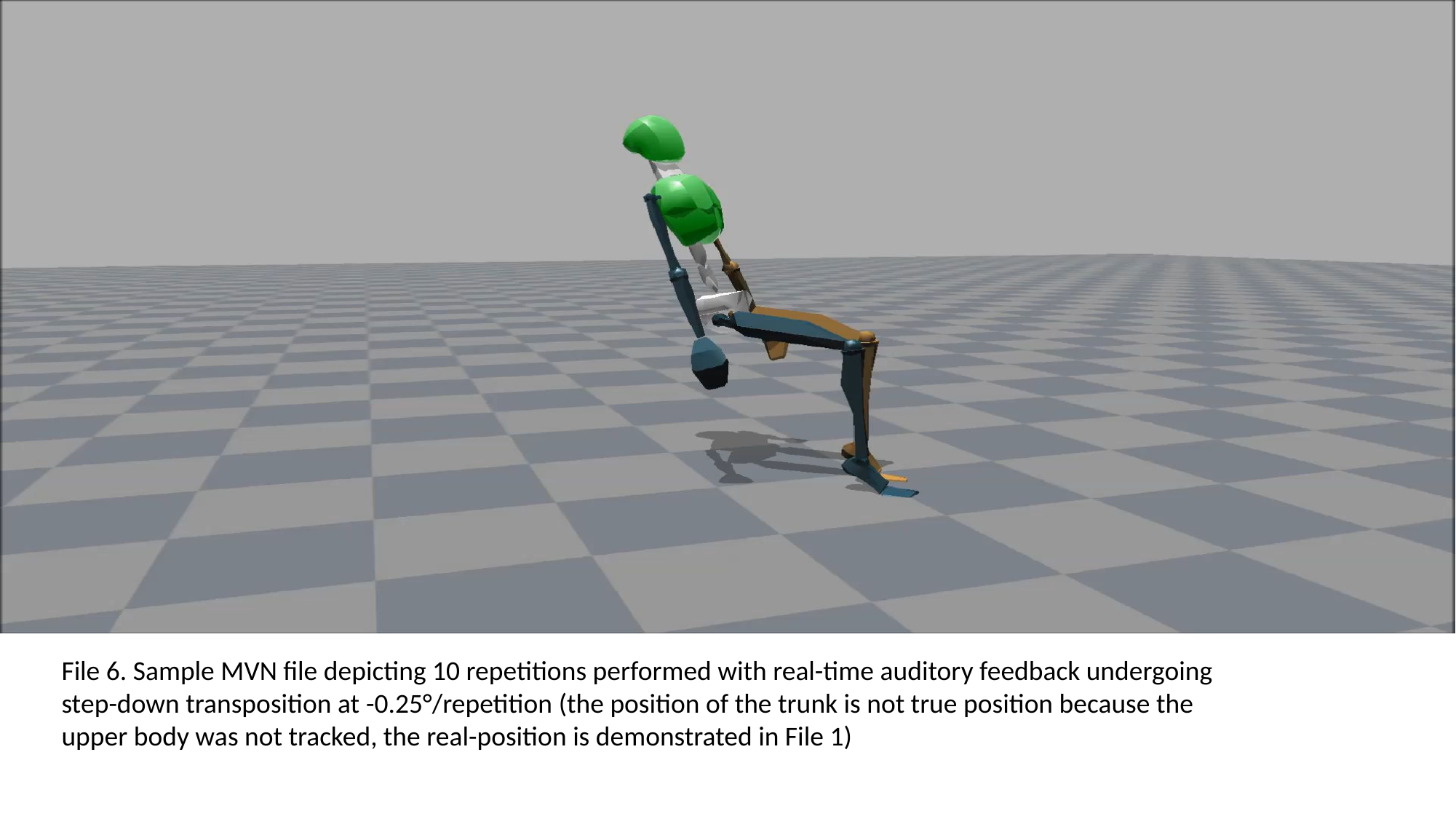

File 6. Sample MVN file depicting 10 repetitions performed with real-time auditory feedback undergoing step-down transposition at -0.25°/repetition (the position of the trunk is not true position because the upper body was not tracked, the real-position is demonstrated in File 1)

## Slide 8
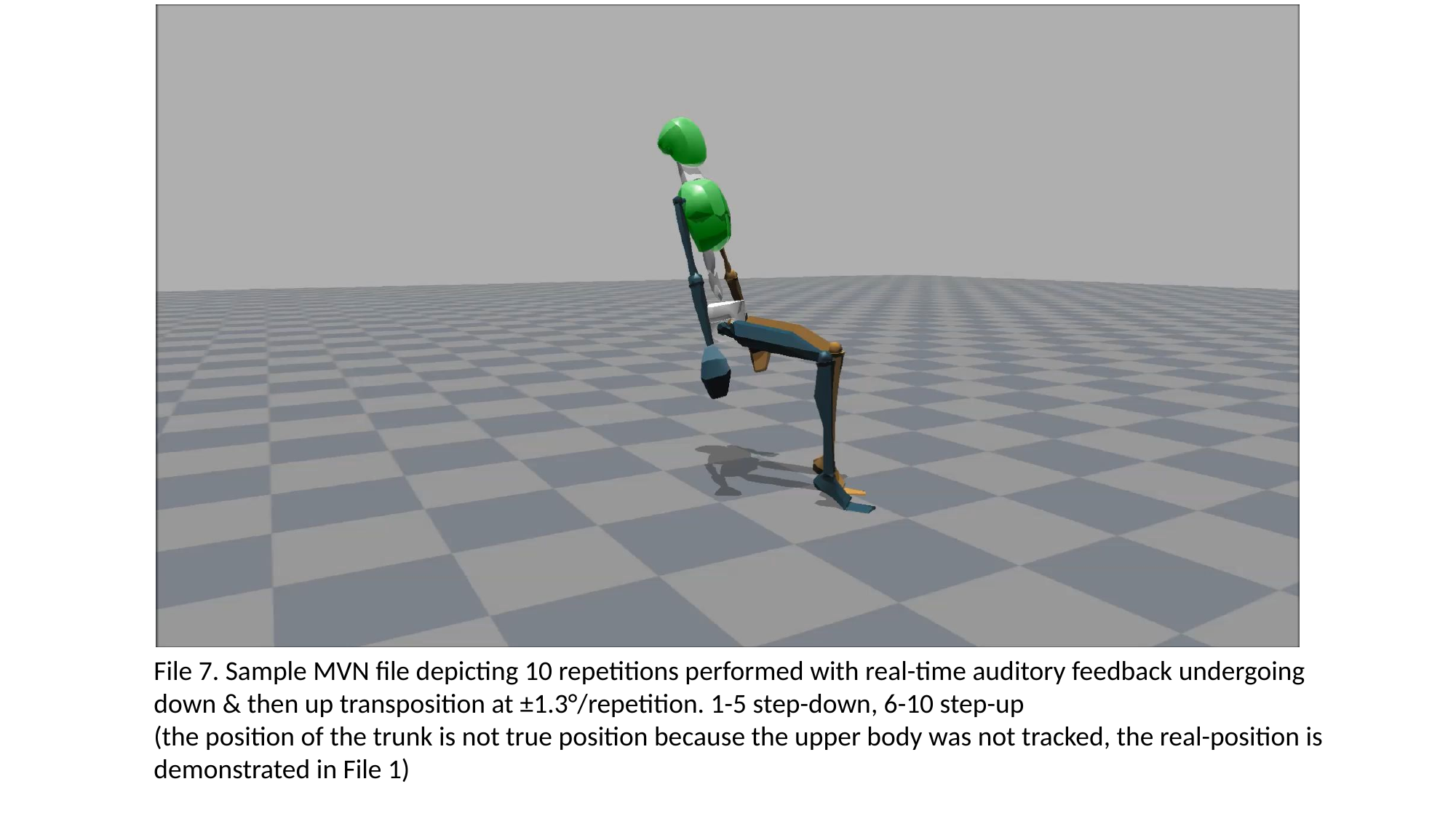

File 7. Sample MVN file depicting 10 repetitions performed with real-time auditory feedback undergoing down & then up transposition at ±1.3°/repetition. 1-5 step-down, 6-10 step-up
(the position of the trunk is not true position because the upper body was not tracked, the real-position is demonstrated in File 1)
